# Supplementary material for: Comparative single-nucleus RNA-seq analysis revealed localized and cell type-specific pathways governing root-microbiome interactions
Source: Nat Commun. 2025 Apr 3;16:3169. doi: 10.1038/s41467-025-58395-0 (PMC11965305; doi:10.1038/s41467-025-58395-0)
Supplement: Supplementary file 1 — Supplementary information [file 41467_2025_58395_MOESM1_ESM.pdf]

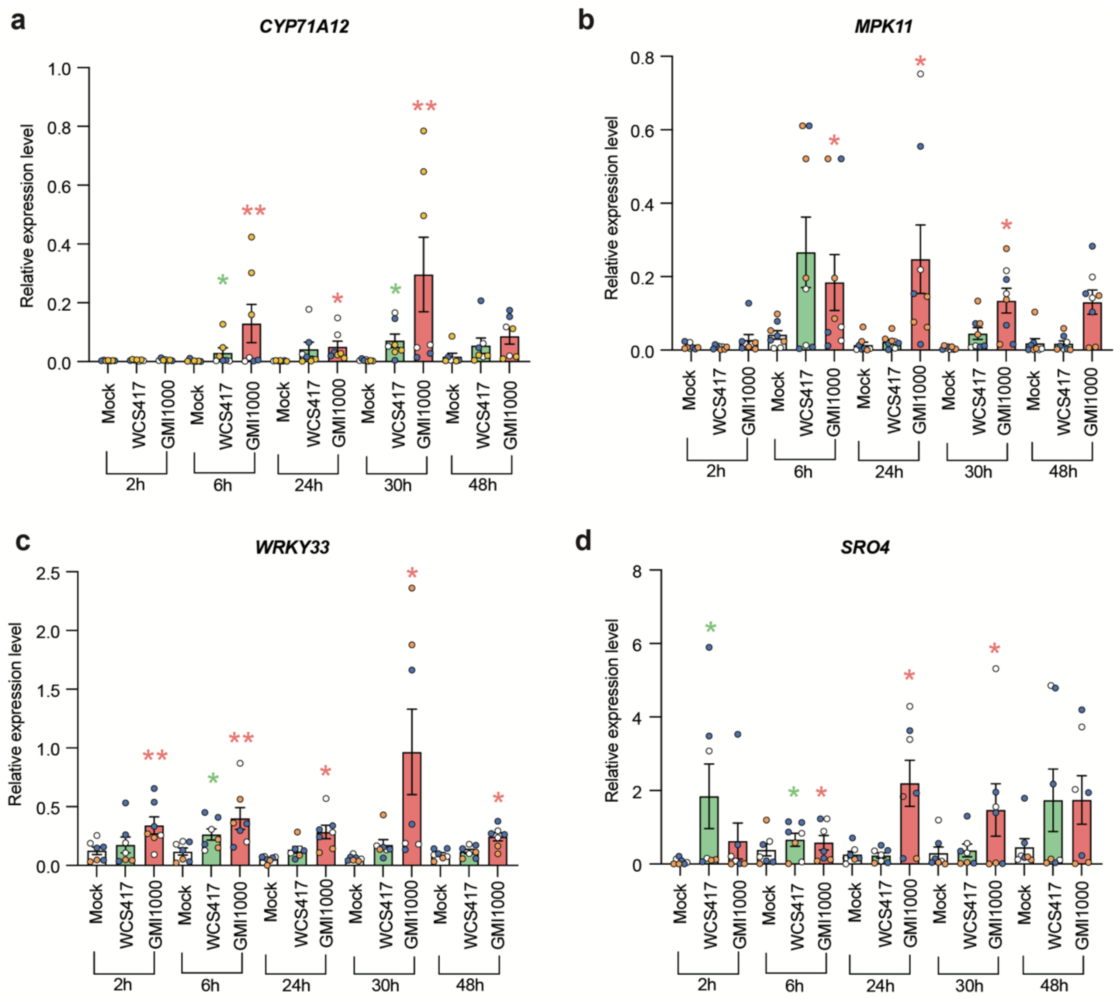

Supplementary Fig. 1 Time-series detection of microbe responsive genes in roots by qRT-PCR. a-d, Immune related *CYP71A12*, *MPK11* and *WRKY33* showed stronger responsiveness to GMI1000, while *SRO4* (a previously reported beneficial WCS417 responsive gene) **d**,showed high responsiveness to WCS417. For MPK11, n=8; for CYP71A12, WRKY33, and SRO4 n=7 samples. Data from 3 independent experiments. The expression of the *EIF4A* gene was used as an internal control. Results are presented as mean ± stand error of the mean (SEM). Asterisks represent the significant (*P<0.05; **P<0.01, two-sided Student's T-test) differences between the bacteria-inoculated group and the $MgSO_4$ group (Mock).

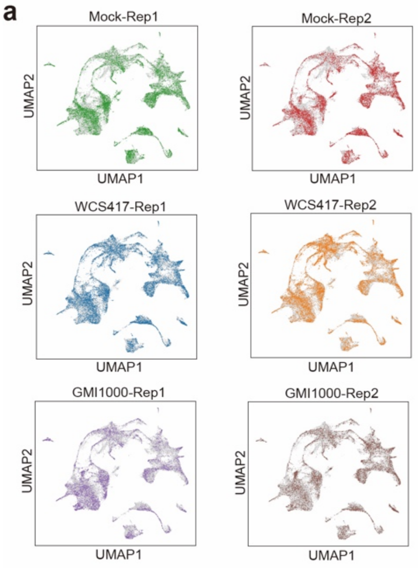

b

Replicates	Spearmen Correlation
Mock Rep1 & Rep2	0.98
WCS417 Rep1 & Rep2	0.98
GMI1000_Rep1 & Rep2	0.97

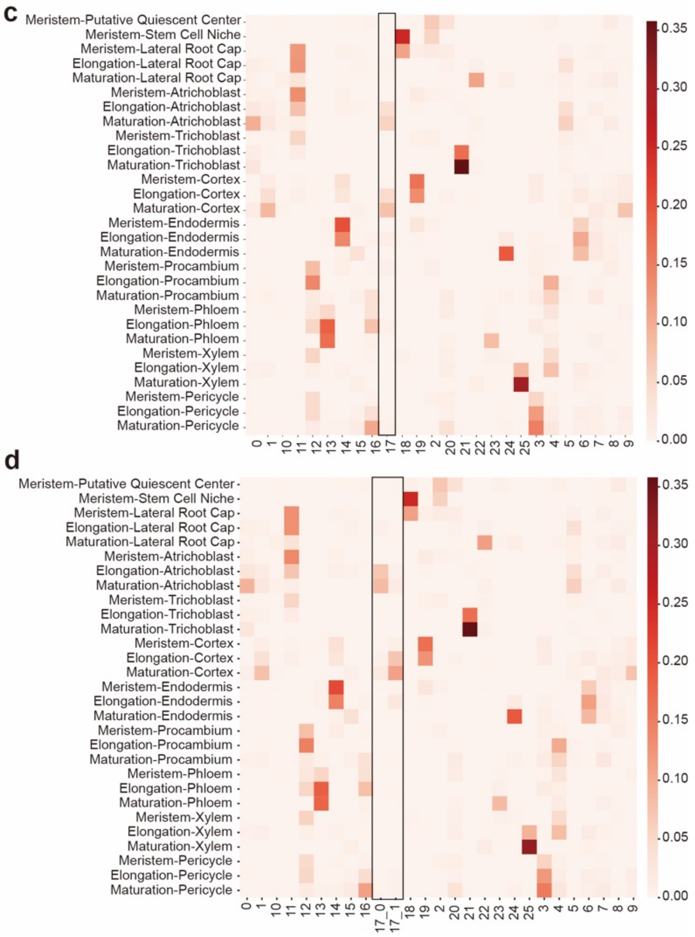

Supplementary Fig. 2 Integration and annotation details of the snRNA-seq data. **a**, Visualization of the six snRNA-seq libraries and their integrated results with final UMAP, which shows high similarities of cell compositions among different samples. **b**, Biological replicate samples show high group consistency with Spearman correlation higher than 0.97. **c,** Intersection of Union (IOU) analysis of the cluster marker genes from our snRNA-seq atlas (Leiden resolution = 0.6) and a comprehensive *Arabidopsis* root scRNA-seq atlas. The cluster marker genes were called with CELLEX (CELL-type EXpression-specificity, v1.2.2) and kept the genes with enrich score > 0.8. **d** Subclusters from cluster 17 were further divided into 17_0 and 17_1, because of a mixed expression of marker genes from distinct cell types.

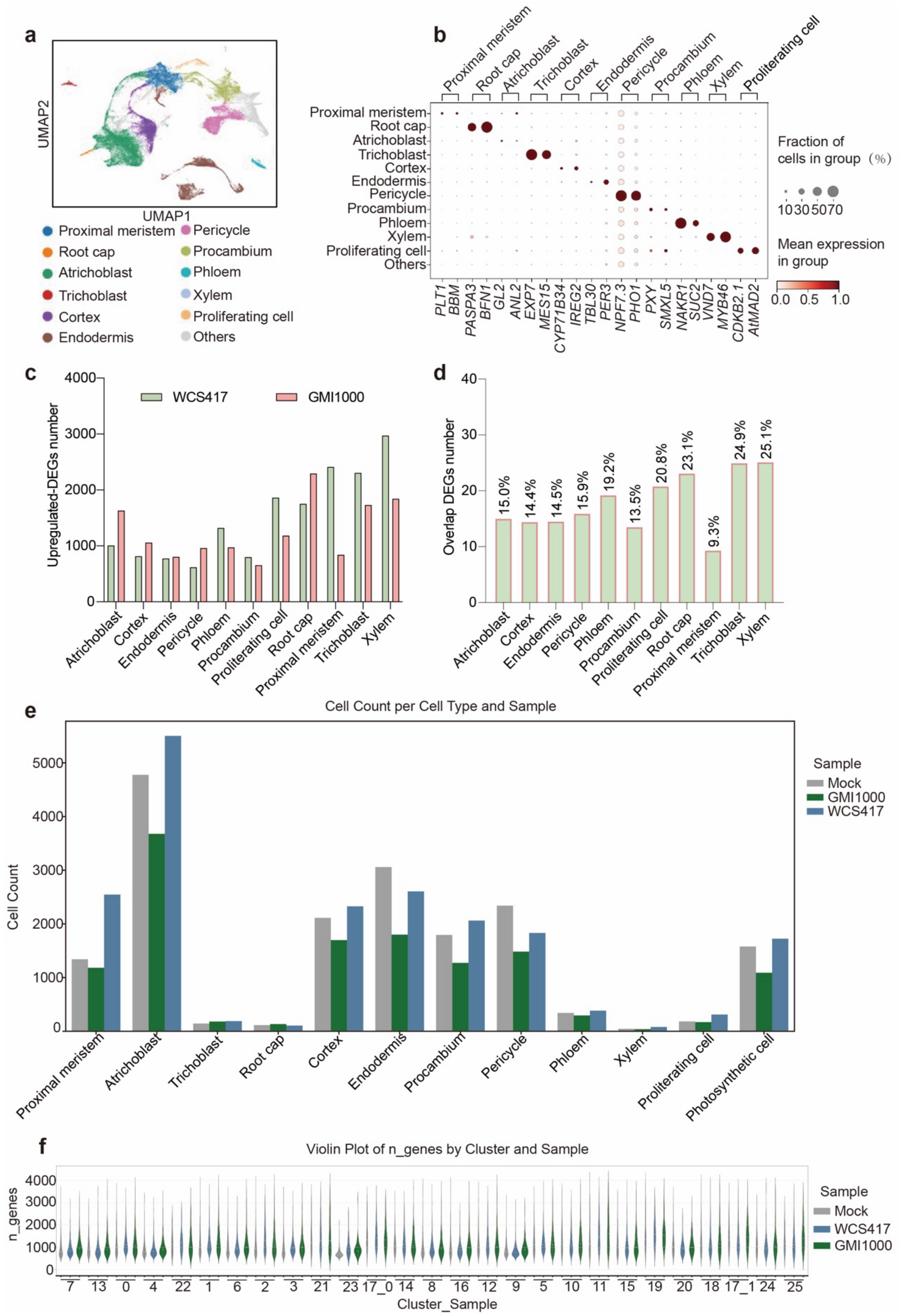

Supplementary Fig. 3 Overview of the d`istribution of DEGs in 11 merged major root cell types. a, UMAP visualization of 11 major root cell types in our snRNA-seq data. **b,** Dotplot showing the expression patterns of selected cell type-specific marker genes. **c,** The number of DEGs in each major root cell type in response to beneficial or

pathogenic microbes. **d,** The ratios of overlapped genes between WCS417- and GMI1000- up-regulated DEGs in different cell types. **e,** The number of cells per merged cluster (11 clusters) for each condition (mock, WCS417, and GMI1000). **f,** The number of genes per sub-cluster (27 clusters) for each treatment. The inner box in the violin plot shows the median (horizontal white bar), 25th (bottoms of boxes), and 75th (tops of boxes) quartiles range (QR), and non-outlier data value (upper and lower whiskers) of the number of genes in each cluster.

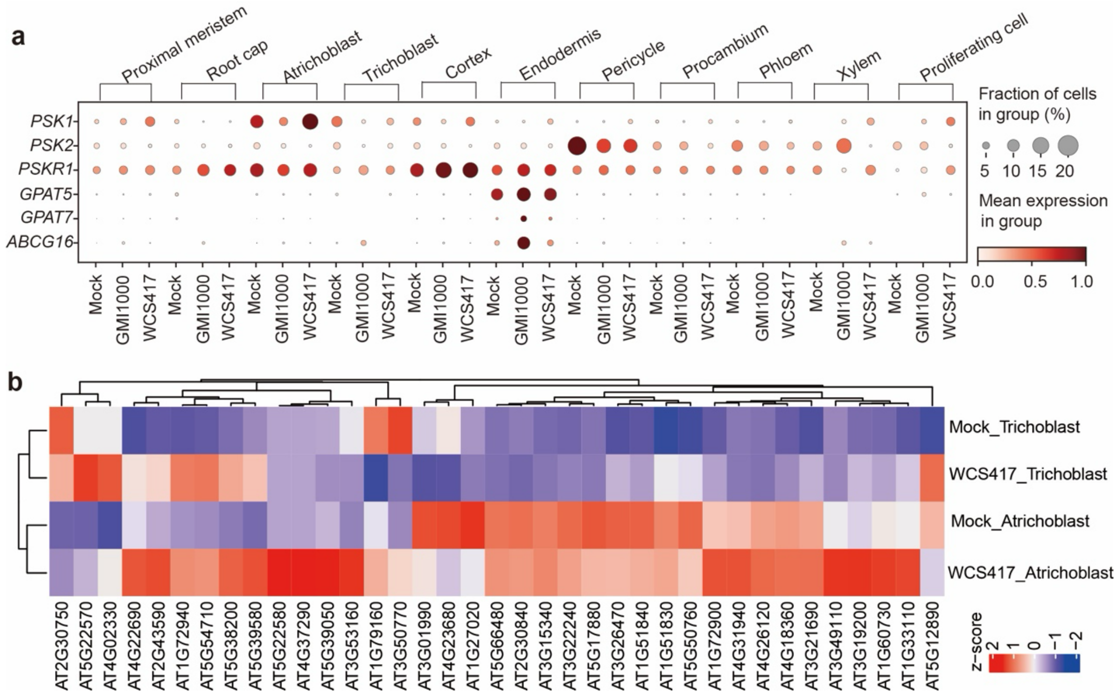

Supplementary Fig. 4 Examination of the expressions of previously reported root-microbe interaction-related genes. a, The expression levels of the *PSKs-PSKR1* genes and several endodermis barrier formation-related genes. **b,** The expression levels of immune-related genes show different expression levels in trichoblast and atrichoblast cells reported in the previous study.

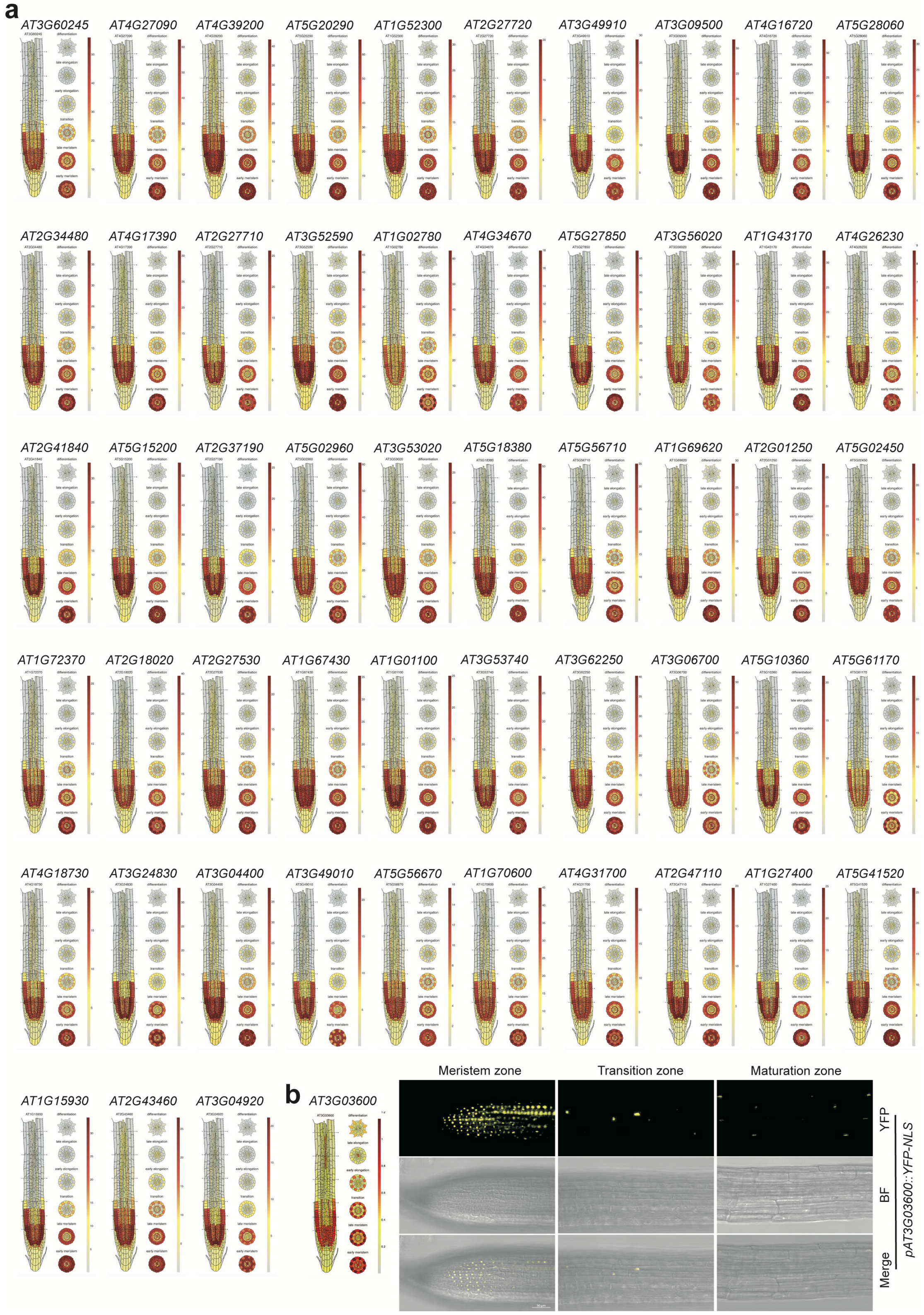

Supplementary Fig. 5 Ribosome-related genes are expressed specifically in the proximal meristem. a, The top 53 ribosome-related genes show proximal meristem-specific expression in the root cell atlas. **b,** Ribosome-related gene *AT3G03600* shows proximal meristem-specific expression in root cell atlas (left) and *in vivo* (right). Confocal images showing the expression pattern of *pAT3G03600::YFP-NLS* of 10-day-

old seedling roots**.** Scale bars, 50 μm.

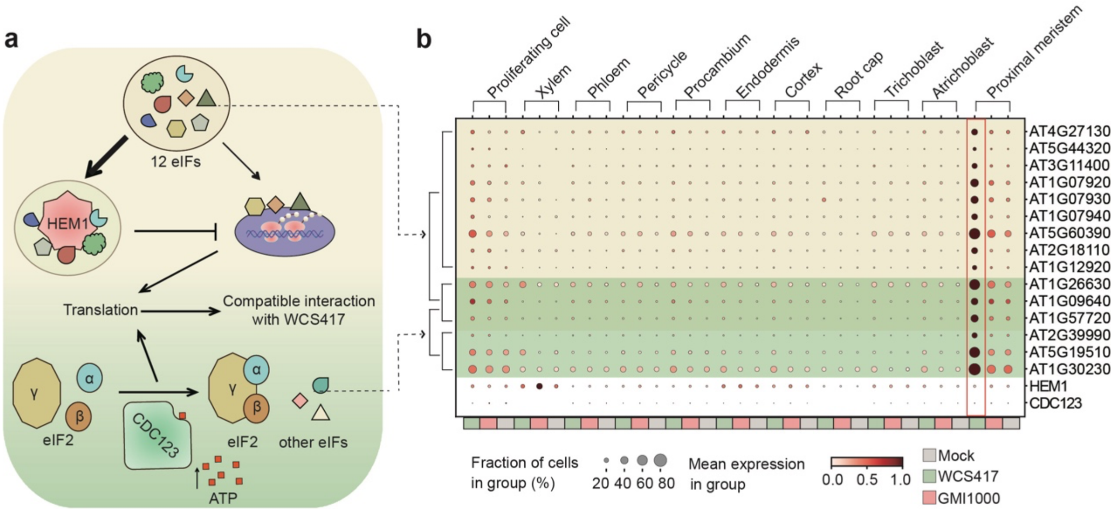

Supplementary Fig. 6 Diverse HEM1 and CDC123's regulatory targets show cell type specifical expression responsiveness to WCS417 in the proximal meristem (belonging to cluster D genes). a, Schematic model showing that HEM1 and CDC123 are involved in transcriptional regulation by regulating key ribosome assembly and function-related genes. **b,** The expression levels of the HEM1- and CDC123-regulated ribosome-related genes that belong to cluster D.

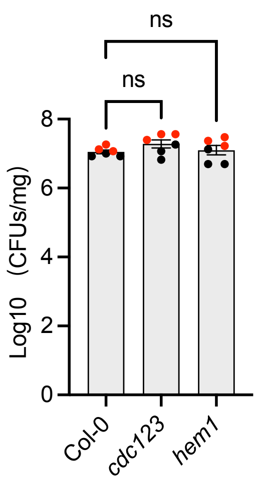

Supplementary Fig. 7 The *cdc123* and *hem1* mutants do not affect the colonization level of WCS417. The colonization levels of WCS417 on roots of Col-0, *cdc123,* and *hem1*. We mixed WCS417 (final concentration of $OD_{600}=10^{-5}$) into half MS (no sucrose, 1% agar) medium before pouring plate (about 50 degree medium). Four roots were harvested at 3 weeks after sowing before calculating CFUs. $n = 6$ from two independent experiments, and different colors indicate data points from different individual experiments. Student's two-tailed T-test was used for comparison.

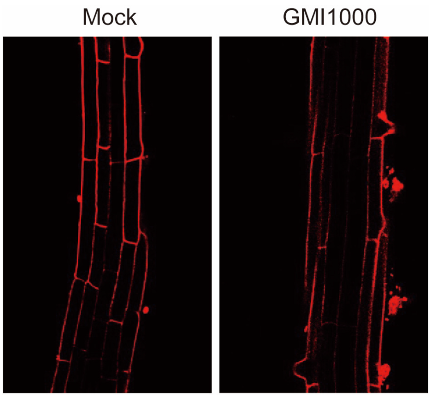

Supplementary Fig. 8 Confocal imaging of PI staining signal in root maturation zone with and without GMI1000 inoculation. Seven-day-old roots were inoculated with liquid half MS without sucrose medium(mock, left), and GMI1000 (OD_{600}=0.05, right) for 6 hours. Confocal images show no significant cell death (intense PI signal inside the cell) in the root maturation zone. The experiment was repeated three times with consistent phenotypes.

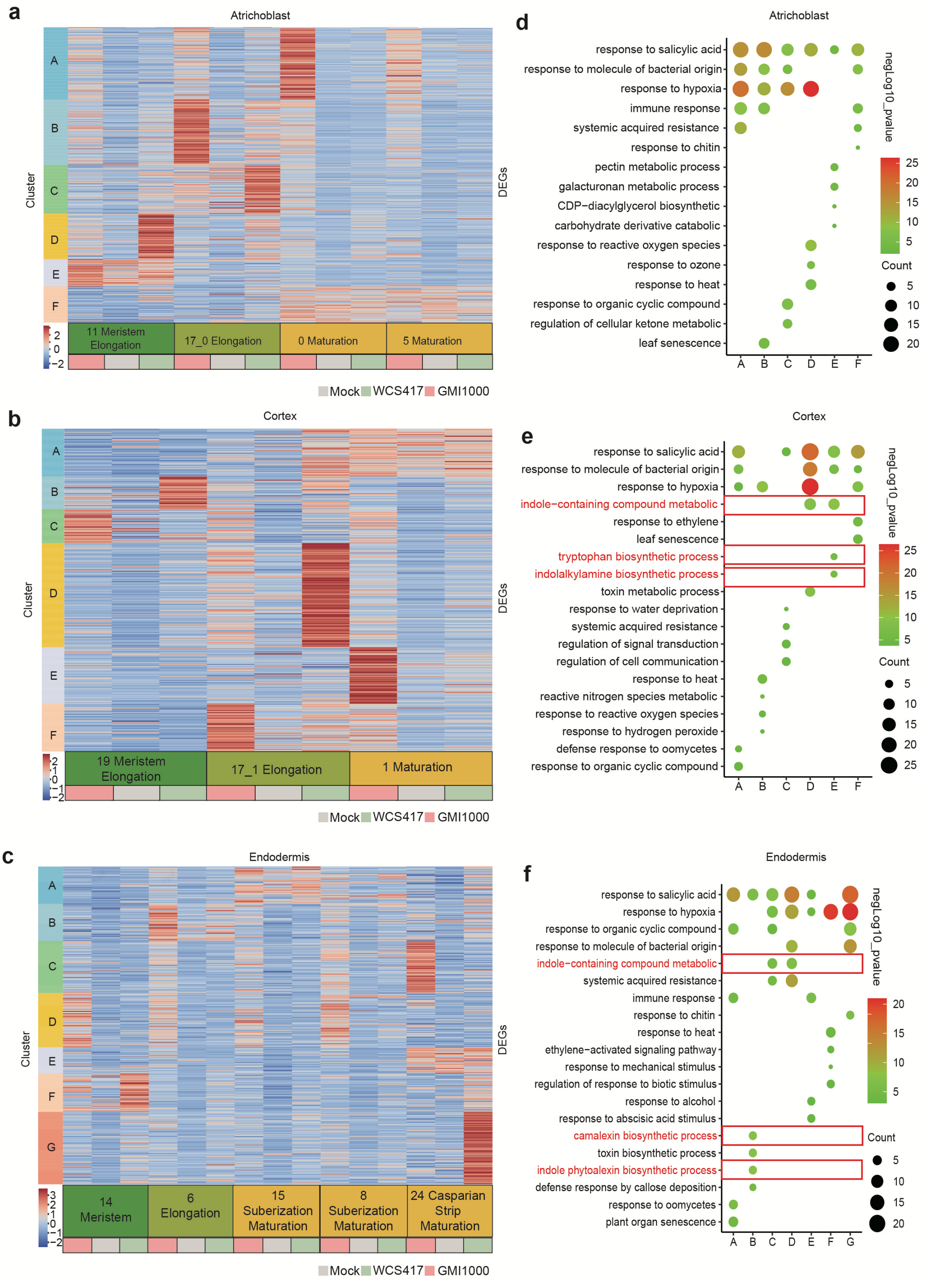

Supplementary Fig. 9 K-means clustering and GO enrichment analysis for core immune responsive genes across cell types from different developmental stages. a-c, Heatmap and K-means clustering of the core immune responsive genes from different cell types (Atrichoblast, cortex, and endodermis) with different developmental stages (maturation, elongation, and meristem). The heatmap is colored by normalized Z-scores. **d-f,** GO terms enriched in each cluster from maturation, elongation, and

meristem zone of the root atrichoblast, cortex, and endodermis cells, respectively. The X-axis letters from d-f correspond to each cluster in a-c, respectively. Red boxes indicate phytoalexin biosynthesis-related GO terms which are routinely enriched in gene clusters show high expression in root maturation cells.

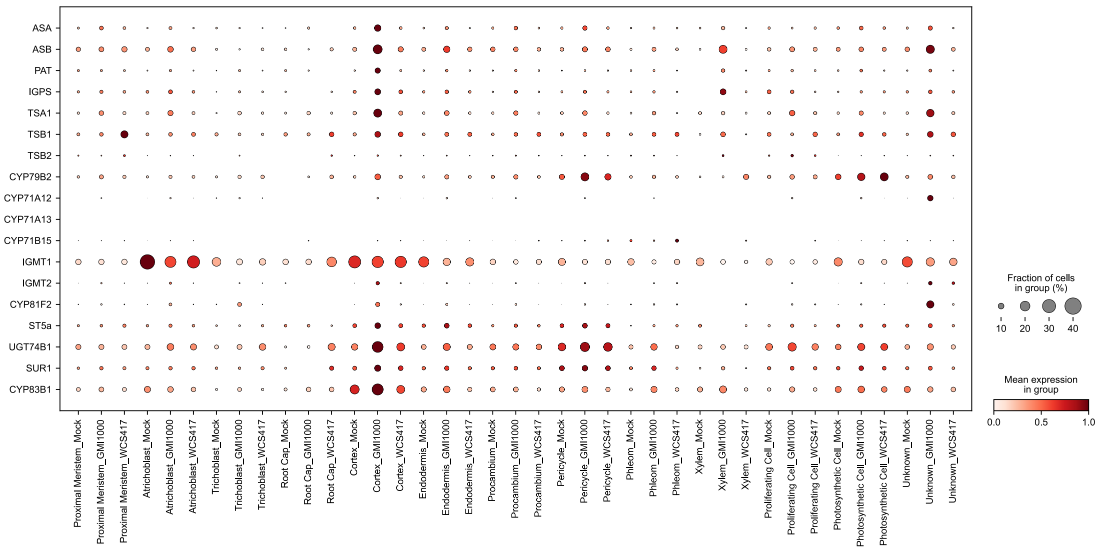

Supplementary Fig. 10 IGS pathway genes show enriched expression and responsiveness to *Ralstonia* in cortex cells. Dotplot showing the expression levels of IGS-related genes from different cell clusters.

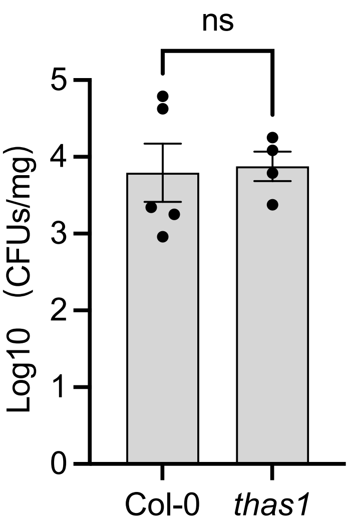

Supplementary Fig. 11 THAS1 does not directly affect GMI1000 resistance in mono-association. The colonization levels of GMI1000 in the leaves (shoots) were measured from Col-0, *thas1* mutant at 3 days post-inoculation. GMI1000 was inoculated on root tips for all seedlings grown on solid MS plates. 5 μL GMI1000 was inoculated on 8-day-old seedlings at a final concentration of OD_{600}=0.0001. Four shoots were mixed as a biological replicate at 3 days post-inoculation for CFU measuring. Student's T-test (two sides) was used for comparison(Col-0, n = 5; *thas1*, n = 4).

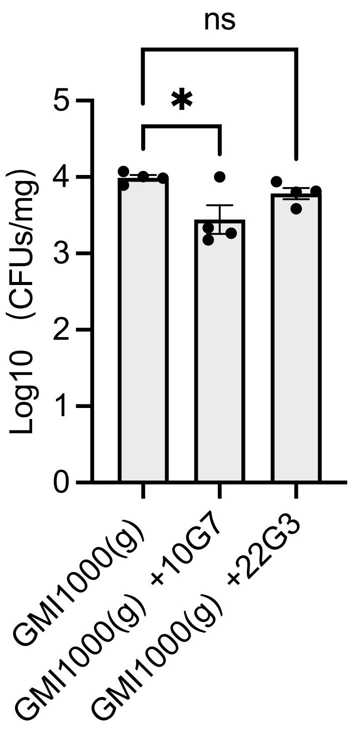

Supplementary Fig. 12 Commensal outcompeting cannot fully explain the biocontrol ability towards GMI1000. The colonization levels of GMI1000g in commensal-GMI1000 community. GMI1000 was transformed into a gentamicin resistance vector (pBB-GFP) and was renamed GMI1000g, while 22G3/10G7 has no gentamicin resistance. Asterisks represent the significant (*$P<0.05$) differences in the colonization levels of GMI1000g between GMI1000 single inoculation and 10G7 co-inoculation, as determined by Student's T-test (two-sided) ($n = 4$).

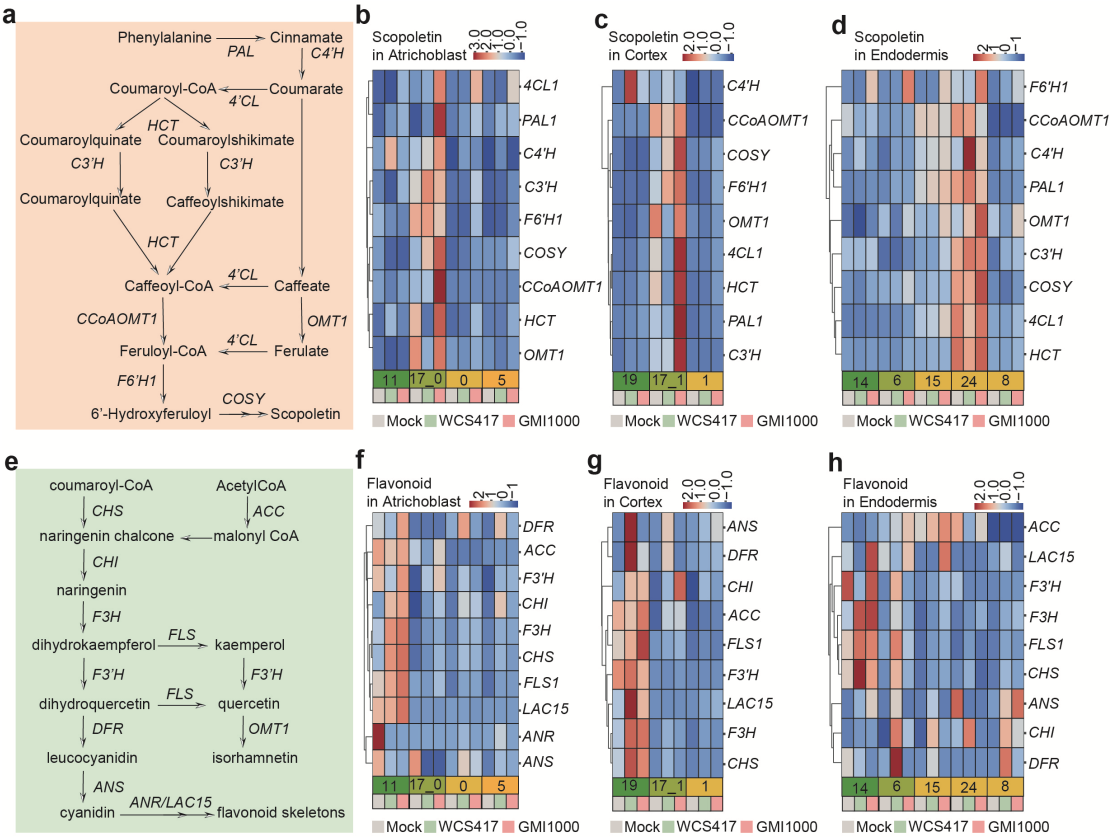

Supplementary Fig. 13 Examination of the expression patterns of scopoletin and flavonoid biosynthesis genes. a, Overview of the scopoletin biosynthesis pathway; **b-d,** Heatmap illustration of the *Z*-score normalized expression levels of scopoletin biosynthesis-related genes from different cell types (atrichoblast, cortex, and endodermis) in different developmental stages; **e,** Overview of the flavonoid biosynthesis pathway; **f-h,** Heatmap illustration of the *Z*-score normalized expression levels of flavonoid biosynthesis-related genes from different cell types (atrichoblast, cortex and endodermis) in different developmental stages.

Supplementary Table 1. Libraries informaiton

Sample ID	Mock		WCS417		GMI1000	
**Replicates**	**1**	**2**	**1**	**2**	**1**	**2**
	**Cellranger Result**					
**10xGenomics Kit**	v3	v3	v3	v3	v3	v3
**Cell Ranger Version**	6.0.0	6.0.0	6.0.0	6.0.0	6.0.0	6.0.0
**Depth (Number of Reads)**	132,910,861	191,664,898	165,195,340	211,622,398	139,231,135	217,593,142
**Cell Ranger V3.0.1 Raw Cells**	14,692	8,758	11,924	11,149	8,106	7,284
**Mean Reads per Cell**	9,046	21,885	13,854	18,981	17,176	29,873
**Median Genes per Cell**	696	1,047	922	1,040	826	1,184
**Valid Barcodes**	95.4%	94.4%	95.8%	94.0%	94.7%	94.8%
	**Quality Control**					
**QC standard**	**700 < Cell Readcount < 4000 & 400< Cell Genecount < 4000 & percent_ct/mt < 0.05**					
**After QC Genomic Gene Count**	24,053	24,682	24,502	24,734	24,298	24,718
**After QC Cell Count**	10,122	8,349	10,586	9,967	6,823	6,859
**After QC Cell Genomic Genecount Median**	842	1,066	981	1,081	1,228	1,594
**After QC Cell Readcount Median**	1,118	1,435	1,392	1,445	902	1,201

Supplementary Table 2. Well-characterized cell type marker genes for validation used in this study.

Cell Type	Gene Name	Gene ID	Reference DOI
Proximal meristem	*PLT1*	AT3G20840	https://doi.org/10.1038/nature06206
Proximal meristem	*BBM*	AT5G17430	https://doi.org/10.1038/nature06206
Root cap	*PASPA3*	AT4G04460	https://doi.org/10.1016/j.cub.2014.03.025
Root cap	*BFN1*	AT1G11190	https://doi.org/10.1038/s41477-018-0212-z
Atrichoblast	*GIR1*	AT5G06270	https://doi.org/10.1126/science.1146265
Atrichoblast	*GL2*	AT1G79840	https://doi.org/10.1371/journal.pgen.1002446
Atrichoblast	*MYB30*	AT3G28910	https://doi.org/10.1126/science.adf4721
Atrichoblast	*ANL2*	AT4G00730	https://doi.org/10.1016/j.devcel.2019.02.022
Trichoblast	*PRP3*	AT3G62680	https://doi.org/10.1104/pp.109.140905
Trichoblast	*EXP7*	AT1G12560	https://doi.org/10.1105/tpc.006437
Trichoblast	*MES15*	AT1G69240	https://doi.org/10.1016/j.devcel.2019.02.022
Cortex	*IREG2*	AT5G03570	https://doi.org/10.1016/j.devcel.2019.02.022
Cortex	*CYP71B26*	AT3G26290	https://doi.org/10.1016/j.chom.2020.11.014
Cortex	*CYP71B34*	AT3G26300	https://doi.org/10.1016/j.devcel.2019.02.022
Endodermis	*PER3*	AT1G05260	https://doi.org/10.1016/j.cell.2013.02.045
Endodermis	*TBL30*	AT2G40160	https://doi.org/10.1016/j.devcel.2019.02.022
Endodermis	*LTPG20*	AT3G22620	https://doi.org/10.1016/j.devcel.2019.02.022
Endodermis	*KCS1*	AT1G01120	https://doi.org/10.1073/pnas.1522466113
Pericycle	*NPF7.3*	AT1G32450	https://doi.org/10.1073/pnas.2013305117
Pericycle	*PHO1*	AT3G23430	https://doi.org/10.1105/tpc.112.096636
Procambium	*PXY*	AT5G61480	https://doi.org/10.1242/dev.044941
Procambium	*ATHB-8*	AT4G32880	https://doi.org/10.1046/j.1469-8137.2003.00769.x
Procambium	*ATHB-15*	AT1G52150	https://doi.org/10.1093/pcp/pcg164
Phloem	*NAKR1*	AT5G02600	https://doi.org/10.1105/tpc.110.080010
Phloem	*SUC2*	AT1G22710	https://doi.org/10.1105/tpc.110.080010
Xylem	*VND7*	AT1G71930	https://doi.org/10.1111/j.1365-313X.2008.03533.x
Xylem	*MYB46*	AT5G12870	https://doi.org/10.1105/tpc.107.053678
Photosynthetic cell	*LHCA1*	AT3G54890	https://doi.org/10.3390/ijms18112352
Photosynthetic cell	*RBCS3B*	AT5G38410	https://doi.org/10.1093/jxb/err434
Proliferating cell	*CDKB2.1*	AT1G76540	https://doi.org/10.1016/j.devcel.2021.02.021
Proliferating cell	*AtMAD2*	AT3G25980	https://doi.org/10.1016/j.devcel.2021.02.021

Supplementary Table 3. Primer used in this study

Primer	Sequence	Application
EIF4A-F	5'-GCAGTCTCTTCGTGCTGACA-3'	qRT
EIF4A-R	5′-TGTCATAGATCTGGTCCTTGAA-3′	qRT
CYP71A12-F	5'-GATTATCACCTCGGTTCCT-3'	qRT
CYP71A12-R	5'-CCACTAATACTTCCCAGATTA-3'	qRT
WRKY33-F	5'-AGCAAAGAGATGGAAAGGGGACAA-3'	qRT
WRKY33-R	5'-GCACTACGATTCTCGGCTCTCTCA-3'	qRT
SRO4-F	5'-TTGGAGCACACACATGAACA-3'	qRT
SRO4-R	5'-CCAAGGAGATTTCGGGTTTT-3'	qRT
MPK11-F	*5′-CATTACAGTCGATGAAGCCTTG-3′*	qRT
MPK11-R	*5′-TGATGTTCTCTTCCGTCAATGA-3′*	qRT
THAS1 promoter F	*5′-tgaccatgattacgaattcctgacgacacatgatgtgac-3′*	PCR
THAS1 promoter R	*5′-tttgtaaggttgctctaagtttacttggacaaggaggac-3′*	PCR
CYP71A12 promoter F	*5′-ccatgattacgaattcgttctaccagcagccttgc-3′*	PCR
CYP71A12 promoter R	*5′-cccttgctcaccatggttcttgaatattgctcatgtatgaaag-3′*	PCR
AT3G03600 promoter F	*5′-gaccatgattacgaattcaattgacagtgacagtcaaa-3′*	PCR
AT3G03600 promoter R	*5′-cttgctcacCATggagttcgtcgtcg-3′*	PCR
