# Supplementary material for: Comparative single-nucleus RNA-seq analysis revealed localized and cell type-specific pathways governing root-microbiome interactions
Source: Nat Commun. 2025 Apr 3;16:3169. doi: 10.1038/s41467-025-58395-0 (PMC11965305; doi:10.1038/s41467-025-58395-0)
Supplement: Supplementary file 2 — Description of Additional Supplementary Files [file 41467_2025_58395_MOESM2_ESM.pdf]

Description of Additional Supplementary Files:

Supplementary Data 1: Representative marker genes generated by CELLEX from our data.

Supplementary Data 2: Differentially expressed genes (DEGs) in each root cell type between mock and different microbe treatments.

Supplementary Data 3: Gene expression levels (CPM) for in all clusters of different samples and treatments.

Supplementary Data 4: Information of Oxalobacteraceae strains used in this study
