# Supplementary material for: Comparative single-nucleus RNA-seq analysis revealed localized and cell type-specific pathways governing root-microbiome interactions
Source: Nat Commun. 2025 Apr 3;16:3169. doi: 10.1038/s41467-025-58395-0 (PMC11965305; doi:10.1038/s41467-025-58395-0)
Supplement: Supplementary file 7 — Reporting Summary [file 41467_2025_58395_MOESM7_ESM.pdf]

nature portfolio

Corresponding author(s): Yi Song

Last updated by author(s): Feb 9, 2025

Reporting Summary

Nature Portfolio wishes to improve the reproducibility of the work that we publish. This form provides structure for consistency and transparency in reporting. For further information on Nature Portfolio policies, see our Editorial Policies and the Editorial Policy Checklist.

Statistics

For all statistical analyses, confirm that the following items are present in the figure legend, table legend, main text, or Methods section.

n/a	Confirmed	
☐	☒	The exact sample size (*n*) for each experimental group/condition, given as a discrete number and unit of measurement
☐	☒	A statement on whether measurements were taken from distinct samples or whether the same sample was measured repeatedly
☐	☒	The statistical test(s) used AND whether they are one- or two-sided<br>*Only common tests should be described solely by name; describe more complex techniques in the Methods section.*
☒	☐	A description of all covariates tested
☒	☐	A description of any assumptions or corrections, such as tests of normality and adjustment for multiple comparisons
☒	☐	A full description of the statistical parameters including central tendency (e.g. means) or other basic estimates (e.g. regression coefficient) AND variation (e.g. standard deviation) or associated estimates of uncertainty (e.g. confidence intervals)
☒	☐	For null hypothesis testing, the test statistic (e.g. *F*, *t*, *r*) with confidence intervals, effect sizes, degrees of freedom and *P* value noted<br>*Give P values as exact values whenever suitable.*
☒	☐	For Bayesian analysis, information on the choice of priors and Markov chain Monte Carlo settings
☒	☐	For hierarchical and complex designs, identification of the appropriate level for tests and full reporting of outcomes
☒	☐	Estimates of effect sizes (e.g. Cohen's *d*, Pearson's *r*), indicating how they were calculated

Our web collection on statistics for biologists contains articles on many of the points above.

Software and code

Policy information about availability of computer code

Data collection	The number of lateral roots and confocal images were measured and analyzed using Fiji software. Data in Supplementary Figure. 5a was collected from the open-access single-cell data website https://rootcellatlas.org/

Data analysis	Data processing and cell type annotation for snRNA-seq analysis: The raw reads were preprocessed by Cell Ranger (v6.0.0) and aligned to the Arabidopsis TAIR 10 genome, generating the h5 files for each sample. Then we used SCANPY package (v1.8.0) in Python to read the files and used ScDblFinder (v1.10.0) to remove the doublet. After that, we kept only the cells with gene counts larger than 300 and less than 3500, and UMI counts larger than 500 and less than 6000. In addition, cells with more than 0.05% transcripts from mitochondria or chloroplasts were removed. We then adopted Seurat (v. 4.3.0) to integrate the six matrixes following the official guidance (https://satijalab.org/seurat/articles/integration_introduction). We used SCTransform to normalize and call the top 3000 highly variable features for each matrix and selected the shared high variable features for canonical correlation analysis (CCA) to identify the integrating anchors. After that, we used the 'scanpy.pp.neighbors' function to call the neartest-neighbour graph (default parameter) and performed the Leiden algorithm (scanpy.tl.leiden, resolution = 0.6) on the graph for clustering. The Uniform Manifold Approximation and Projection (UMAP) algorithm was used for visualization (scanpy.tl.umap, resolution = 0.2). For annotation, we first used CELLEX (CELL-type Expression-specificity, v1.2.221) to call the cluster-enriched genes from our data and the reference data, and annotated the clusters according to their Intersection of Union level.<br>Microbiome data analysis: Raw sequencing reads of amplicon sequencing were first filtered using fastp v.0.14.172. Adapter sequences and primers were removed using Cutadapt v.4.073. Retained reads were further filtered and taxonomically analyzed using QIIME2 v.2022.2. In brief, DADA2 was used to filter and denoise sequences, remove chimaeras, identify representative sequences, and generate a unique amplicon sequence variants (ASVs) table. ASVs were taxonomically annotated via a pre-trained naive Bayes classifier on the basis of the SILVA database (release 138). Sequences annotated as chloroplasts and mitochondria were considered host contamination and were thus removed. ASVs that were present in more than 3 samples were used for downstream analysis.

For manuscripts utilizing custom algorithms or software that are central to the research but not yet described in published literature, software must be made available to editors and reviewers. We strongly encourage code deposition in a community repository (e.g. GitHub). See the Nature Portfolio guidelines for submitting code & software for further information.

Data

Policy information about availability of data

All manuscripts must include a data availability statement. This statement should provide the following information, where applicable:

- Accession codes, unique identifiers, or web links for publicly available datasets
- A description of any restrictions on data availability
- For clinical datasets or third party data, please ensure that the statement adheres to our policy

All the codes for snRNA-seq analysis in this work can be found at Zhai lab's GitHub site: https://github.com/ZhaiLab-SUSTech/snRNA-seq_microbes.
The snRNA-seq and microbiome data were deposited in the China National Center for Bioinformation database (www.cncb.ac.cn) under PRJCA019084 and PRJCA026420, respectively.

Research involving human participants, their data, or biological material

Policy information about studies with human participants or human data. See also policy information about sex, gender (identity/presentation), and sexual orientation and race, ethnicity and racism.

Reporting on sex and gender	Not applicable in this study!
Reporting on race, ethnicity, or other socially relevant groupings	Not applicable in this study!
Population characteristics	Not applicable in this study!
Recruitment	Not applicable in this study!
Ethics oversight	Not applicable in this study!

Note that full information on the approval of the study protocol must also be provided in the manuscript.

Field-specific reporting

Please select the one below that is the best fit for your research. If you are not sure, read the appropriate sections before making your selection.

☒ Life sciences ☐ Behavioural & social sciences ☐ Ecological, evolutionary & environmental sciences

For a reference copy of the document with all sections, see nature.com/documents/nr-reporting-summary-flat.pdf

Life sciences study design

All studies must disclose on these points even when the disclosure is negative.

Sample size	We did not use statistical models to predetermine sample size.
Data exclusions	No data were excluded.

Replication	For all experiments, including single-cell libraries and wet lab experiments, at least two replicate experiments were conducted. The number of biological replicates is specified in the manuscript.
Randomization	For snRNA-seq and microbiome sampling, the plants were randomly assigned to the different treatment groups.
Blinding	Data collecting is not blind.

Reporting for specific materials, systems and methods

We require information from authors about some types of materials, experimental systems and methods used in many studies. Here, indicate whether each material, system or method listed is relevant to your study. If you are not sure if a list item applies to your research, read the appropriate section before selecting a response.

Materials & experimental systems

n/a	Involved in the study	
☒	☐	Antibodies
☒	☐	Eukaryotic cell lines
☒	☐	Palaeontology and archaeology
☒	☐	Animals and other organisms
☒	☐	Clinical data
☒	☐	Dual use research of concern
☐	☒	Plants

Methods

n/a	Involved in the study	
☒	☐	ChIP-seq
☒	☐	Flow cytometry
☒	☐	MRI-based neuroimaging

Plants

Seed stocks	The Arabidopsis ecotype Col-0 used in this study is maintained in Song's lab. Arabidopsis mutants cdc123 and hem1 were gifted from Dr. Guoyong Xu. All T-DNA insertion lines (including SALK_045401, SALK_043342, SALK_003799, SALK_067603, SALK_102754, SALK_029203, SALK_110811, SALK_119578, SALK_206632, SALK_126907, SALK_035228, SAIL_1231_C04, SALK_124523) were obtained from ABRC.
Novel plant genotypes	The pTHAS1::YFP-NLS and pCYP71A12::YFP-NLS were generated as transgenic lines (for detailed information see Methods section).
Authentication	All T-DNA insertion mutants were validated by PCR.
